# Identification of a Competing Endogenous RNA Network Related to Immune Signature in Lung Adenocarcinoma

**DOI:** 10.3389/fgene.2021.665555

**Published:** 2021-06-03

**Authors:** Ting Zhu, Yong Yu, Jun Liu, Kaiming Ren

**Affiliations:** ^1^Shengjing Hospital of China Medical University, Shenyang, China; ^2^Department of Thoracic Surgery, Shengjing Hospital of China Medical University, Shenyang, China

**Keywords:** integrated analysis, lung adenocarcinoma, ceRNA, overall survival, tumor infiltrating immune cells

## Abstract

**Background:**

The establishment of immunotherapy has led to a new era in oncotherapy. But the signature of immune-related genes (IRGs) in LUAD remains to be elucidated. Here we use integrated analysis to identify IRGs roles in immune signature and detect their relationship with competing endogenous RNA (ceRNA) networks in LUAD progression.

**Methods:**

By analyzing the RNA-seq data from different platforms, we recognized the differentially expressed genes (DEGs) of each platform and screened out the top 20 hub IRGs related to immune responses. Then, we applied the CIBERSORT algorithm to explore the landscape of tumor-infiltrating immune cells (TILs) in LUAD and their connection with hub genes. Next, we predicted and validated the upstream miRNAs and lncRNAs according to their expression and prognostic roles. Finally, we constructed and validated an immune-related ceRNA network by co-expression analysis.

**Results:**

A total of 71 IRGs were identified among 248 DEGs, which play key roles in immune responses. CIBERSORT analysis showed that six hub genes were closely related to TILs, such as SPP1 and naive B cells (*R* = −0.17), TEK and resting mast cells (*R* = 0.37). Stepwise prediction and validation from mRNA to lncRNA, including 6 hub genes, 5 miRNAs, and 9 lncRNAs, were applied to construct a ceRNA network. Ultimately, we confirmed the TMPO-AS1/miR-126-5p/SPP1 and CARD8-AS1/miR-21-5p/TEK as immune-related ceRNA networks in LUAD progression.

**Conclusion:**

We elucidated two immune-related ceRNA networks in LUAD progression, which can be considered as immunotherapy targets for this disease.

## Introduction

Lung cancer (LC) is the leading cause of cancer-related mortality in China, accounting for nearly 20% of all cancer deaths. And lung adenocarcinoma (LUAD) is the most common type of lung cancer, which comprises about 40% of LC cases (Cao and Chen, 2019; Siegel et al., 2019). Despite advances in diagnosis and therapy for LUAD, the outcome of patents remains unsatisfactory. Immunotherapy has emerged as a breakthrough in the treatment of cancer. However, to date, there has been insufficient research on biomarkers related to immune response, which hinders the further application of immunotherapy in LUAD (Chen H. et al., 2019). Therefore, there is a need to find immune-related genes (IRGs) that could be used to select and optimize immunotherapy, and provide therapeutic targets for LUAD.

Non-coding RNAs (ncRNA) are known to be more abundant than mRNAs in human genome, and can regulate mRNA expression at the transcriptional and post-transcriptional levels (Anastasiadou et al., 2018). This function is mainly modulated via miRNA response elements (MREs), with which ncRNAs can interact to establish a competing endogenous RNA (ceRNA) axis (Karreth and Pandolfi, 2013). According to ceRNA hypothesis, ncRNAs act as “sponges” to absorb and bind miRNA, thereby weakening their ability to mRNA and regulating gene expression (Salmena et al., 2011). Moreover, increasing evidence has indicated that the ceRNA axis plays pivotal roles in the formation and progression of multiple types of cancer, including pancreatic cancer, gastric cancer, as well as LUAD (Li et al., 2017; Wang J. et al., 2020; Zu et al., 2020). Notably, the relationship between immune genes and the ceRNA network has been explored in multiple types of cancer, including gastric cancer (Zhang et al., 2020), breast cancer (Liu et al., 2020), and colon cancer (Chang et al., 2020). And some immune-related targets are also involved in ceRNA networks, for instance, OSTN-AS1 in breast carcinoma, FAS in colorectal carcinoma, and LINC00301 in non-small-cell lung cancer (Liu et al., 2019; Chang et al., 2020; Sun et al., 2020). But their relationship in LUAD remains unclear. Therefore, there is a need to clarify the relationship between immune-related targets and the ceRNA network in LUAD.

Against this background, in this study, we performed a comprehensive analysis to identify IRGs and evaluated their roles in tumor-infiltrating immune cells (TILs). Moreover, we predicted and validated the upstream miRNAs and lncRNAs of those genes according to their expression levels and prognostic roles. The functional analysis indicated that these genes play important roles in immune-related processes and pathways. Finally, we constructed and validated an immune-related ceRNA network by co-expression analysis. The obtained results indicated that the IRGs act as a competing network in LUAD progression, which can be considered as an immunotherapeutic target for LUAD.

## Materials and Methods

### Data Selection and Process

To reduce biases caused by small samples and single cohorts, we performed a comprehensive analysis by screening the RNA-seq data of LUAD from the Gene Expression Omnibus (GEO) and the Cancer Genome Atlas (TCGA) databases (Edgar et al., 2002; Weinstein et al., 2013). We searched the keywords “lung adenocarcinoma” in GEO databases, and obtained all the accession numbers of GEO series (GSE). To reduce biases caused by differences in sequencing platforms, we divided all the GSE according to GEO platform (GPL). Then, we selected datasets with the most samples in each platform for subsequent analysis. Inclusion criteria were as follows: (1) human LUAD samples; (2) tumor and non-tumor samples from different platforms; and (3) cohorts with >100 samples. Finally, three platforms’ samples from GEO datasets and one platform’s samples from TCGA database were selected for screening differentially expressed genes (DEGs). The details are as follows: GSE31210 dataset from the GPL570 platform, GSE68465 dataset from the GPL96 platform, GSE43458 dataset from the GPL6244 platform, and TCGA-LUAD dataset from the Illumina platform. The raw data from GEO were obtained and standardized using “RMA” methods and were annotated by the “Bioconductor” package according to their platform (Gautier et al., 2004). The raw count data of LUAD were downloaded from TCGA database and annotated through the Ensembl database (Howe et al., 2020). Stepwisely, all of the raw count data were log2 (x + 1) transformed and normalized using the “LIMMA” package. Then, we screened out DEGs in four groups using the “LIMMA” package with the criteria of | log2FC| > 1, average expression > 1, and adjusted *P*-value < 0.05 (Ritchie et al., 2015).

### Function Enrichment Analysis

We also identified immune genes from the ImmPort database, which contains genes closely involved in immune activity (Bhattacharya et al., 2014). The IRGs were extracted from immune genes and DEGs. To elucidate the potential functions of the IRGs, we performed Gene Ontology (GO) term analysis and Kyoto Encyclopedia of Gene and Genomes (KEGG) pathway enrichment analysis via the DAVID database and the Kobas database (Huang et al., 2009; Xie et al., 2011). We depicted the top 10 most enriched GO terms and KEGG pathways using the “ggplot2” packages with the threshold of *P*-value < 0.05.

### Immune-Related Genes Identification and Assessment

To understand the interactions among IRGs, we constructed a PPI network with a confidence score of ≥0.4 in the STRING database (Version 11.0), which is a widely used tool for analyzing known protein interactions (Szklarczyk et al., 2017). Next, we used CytoHubba, an app in Cytoscape software (Version 3.7.2), which explores key nodes and fragile motifs in the PPI network, to identify the top 20 hub genes according to their connection degree (Shannon et al., 2003). Additionally, we evaluated the expression levels of hub genes by GEPIA database that contain 483 LUAD samples and 347 non-tumor samples combined TCGA with the Genotype-Tissue Expression (GTEx) database (GTEx Consortium, 2015; Tang et al., 2017). We assessed the prognostic roles of hub genes through the Kaplan-Meier (KM) plotter database, which included survival data from 513 LUAD samples, and the OncoLnc database, which included survival data from 492 LUAD samples (Anaya, 2016; Nagy et al., 2018). We also calculated the hazard ratio with the 95% confidence interval and the log-rank *P*-value. *P* < 0.05 was considered to indicate a statistically significant difference. The qualified hub IRGs were identified for subsequent analysis.

### Tumor Infiltrating Estimation

To evaluate the proportion of TILs in LUAD, we assessed 22 types of immune cell using the CIBERSORT algorithm (Chen et al., 2018). Next, we assessed the accuracy of each sample using a *P*-value, with only samples having a CIBERSORT output of *P* < 0.05 being chosen for further analysis. Additionally, we created a violin plot and a correction plot to depict the distribution of immune cells in LUAD. We also determined the expression levels of each type of immune cell to display their correlations. Furthermore, we estimated the expression levels of qualified IRGs and the TIL proportion in each LUAD sample by correlation analysis using the Wilcoxon rank-sum test. Only immune cells with a *P*-value lower than 0.05 were selected for correction.

### Establishing a ceRNA Network

To explore the potential interaction between mRNAs and miRNAs, the TarBase v.8 database, which includes experimentally validated microRNA-target interactions, was applied to predict the upstream miRNAs of the qualified hub IRGs (Karagkouni et al., 2018). Only those miRNAs for which there was strong evidence, including that obtained using luciferase reporter, western blotting, and qPCR assays, were chosen as candidate miRNAs. Next, we evaluated their expression levels in the starbase database and survival roles in the KM plotter database. Additionally, we employed the miRNet database, a widely used tool for miRNA target prediction, to identify lncRNAs potentially binding to miRNAs (Fan et al., 2016). Next, we obtained the RNA-seq data of lncRNAs from TCGA databases to evaluate the expression levels of candidate lncRNAs, and used a KM plotter database to verify their prognostic roles. Moreover, to visualize the interaction among lncRNAs with qualified miRNAs and candidate mRNAs, we constructed a ceRNA network using Cytoscape software. Finally, we validated the connection of ceRNA pairs by co-expression analysis. The details of ceRNA network regulation are clearly shown in the schematic representations.

### Statistical Analysis

We performed all statistical analyses using R software (version 3.6.1) and the bioinformatic tools mentioned above. We used the two-tailed Student’s *t*-test to analyze the relative expression levels of lncRNAs. We evaluated the correlations between RNA expression levels by Pearson’s correlation analysis. We constructed the ceRNA network using Cytoscape software (Version 3.7.2). And we considered that *P*-values lower than 0.05 reflected statistical significance.

## Results

### Screening Differentially Expressed Genes

**FIGURE 1:**
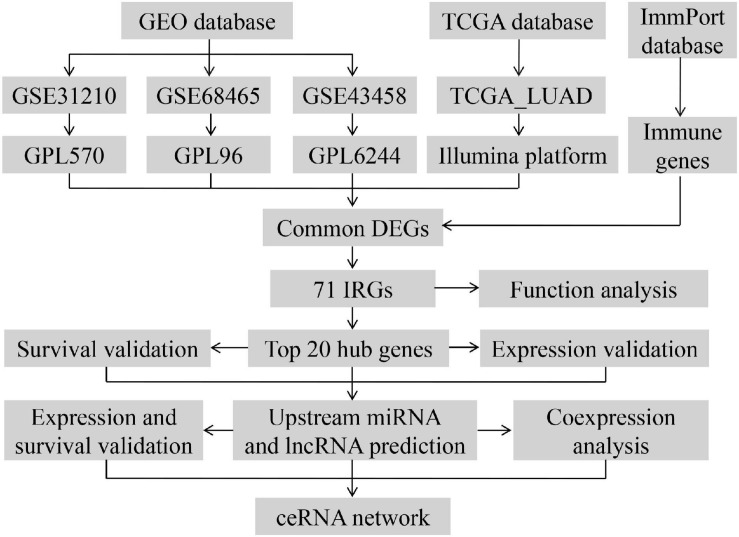
Workflow presenting the process of establishing the immune-related ceRNA network in lung adenocarcinoma.

**FIGURE 2:**
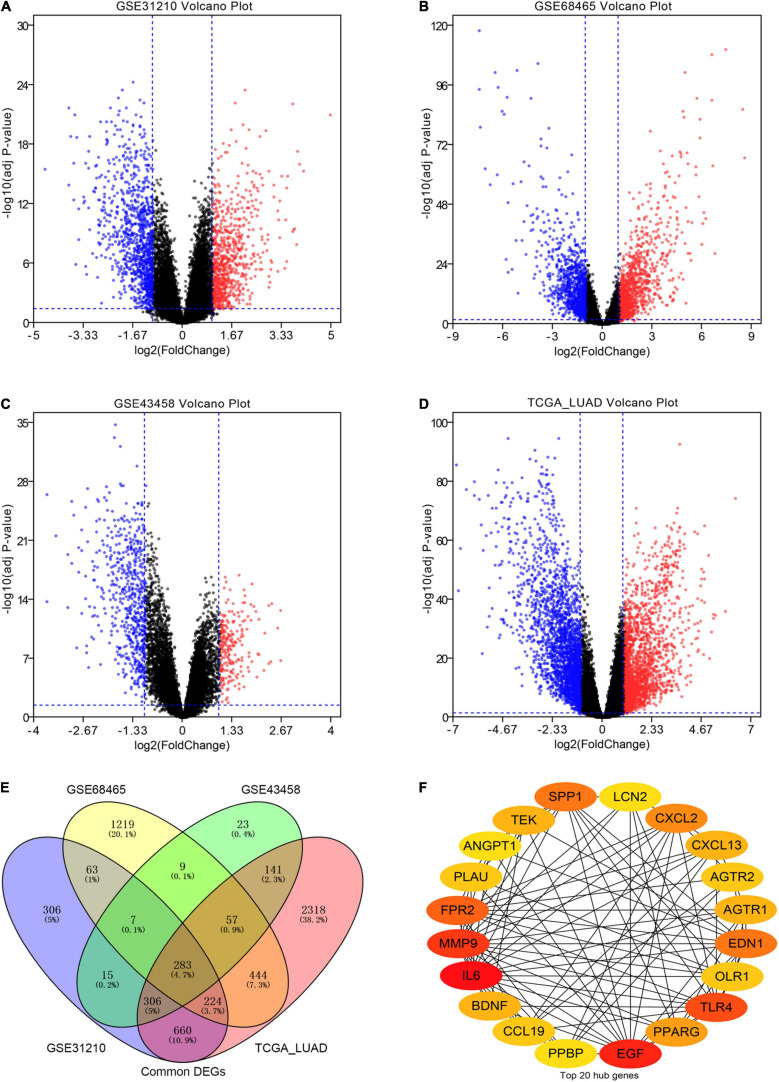
Screening differentially expressed genes (DEGs) in our analysis. **(A–D)** The volcano plots of DEGs in the GSE31210 group, GSE68465 group, GSE43458 group, and TCGA_LUAD group with thresholds of | log2FC| > 1, average expression > 1, and adjust *P*-value < 0.05, respectively. The red dots and blue dots represent the up-regulated and down-regulated DEGs, separately. **(E)** The intersection of DEGs in four groups. **(F)** The top 20 hub immune-related genes (IRGs) in our cohort.

The workflow of our research was depicted in Figure 1. A total of 818 LUAD patients were enrolled from the GEO database and 594 patients from TCGA database. We divided all data into four groups according to their platform, including 226 LUAD samples and 20 non-tumor samples in GPL570, 80 LUAD samples, and 30 non-tumor samples in GPL6244, 443 LUAD tissues, and 19 non-tumor in GPL96, 535 LUAD tissues, and 59 non-tumor in Illumina platform. Then, we identified the DEGs in the four groups using the following thresholds: | log2FC| > 1, average expression > 1, and adjusted *P*-value < 0.05. There were a total of 1,864 DEGs in the GSE31210 group, 2,306 DEGs in the GSE68465 group, 841 DEGs in the GSE43458 group, 4,433 DEGs in the TCGA_LUAD group, and 283 DEGs common to all four groups (Figures 2A–E and Supplementary Table 1). There were a total of 71 IRGs among these 283 common DEGs and 1,794 IRGs, as shown in Supplementary Figure 1A and Supplementary Table 1, which were chosen for subsequent analysis.

### Function Analysis for Immune-Related Genes

We performed the GO term and KEGG pathway analyses to determine the functional roles of the 71 IRGs. A total of 173 GO terms and 14 KEGG pathways were primarily enriched for those IRGs, including 130 terms in the biological process (BP) category, 16 terms in the cellular component (CC) category, and 27 terms in the molecular function (MF) category. We present the top ten BP, CC, and MF terms, and KEGG pathways in Supplementary Figures 2A–D. Briefly, the GO terms indicated that the IRGs were most enriched in immune response, inflammatory response, innate immune response, and cytokine activity. Additionally, several immune-related pathways were detected in the KEGG pathway analysis, for example, the cytokine receptor interaction pathway, HIF-1 signaling pathway, TNF signaling pathway, and Ras signaling pathway. Overall, the results from the functional enrichment analysis indicated that the IRGs were closely involved in the immune response of LUAD.

### Screening and Validating Hub Genes

**FIGURE 3:**
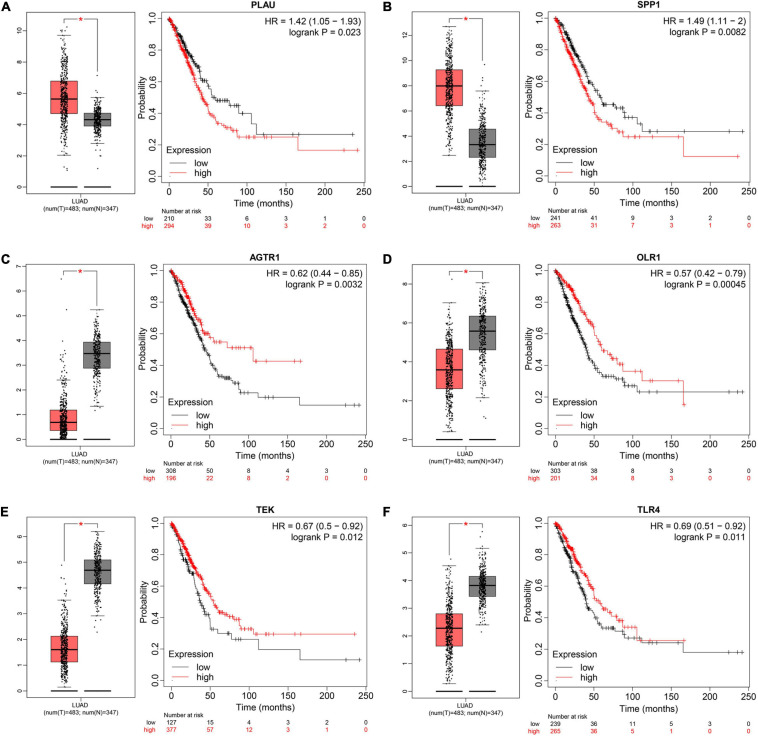
Screening and validating the expression value and prognostic roles of hub IRGs in LUAD. **(A–F)** Validating expression roles and prognosis values of PLUA, SPP1, AGTR1, OLR1, TEK, and TLR4 through GEPIA and KM plotter, respectively (*P < 0.05).

To understand the mutual interaction among IRGs, we constructed a PPI network, as shown in Supplementary Figure 1B. We calculated the node degree of the PPI network using cytoHubba tools from Cytoscape software and identified the top 20 hub genes of IRGs, as shown in Figure 2F. Subsequently, we applied GEPIA to assess the expression levels of the hub IRGs, while we used the KM plotter database and OncoLnc database to evaluate the prognostic roles of the hub IRGs. For the hub IRGs, 7 genes were upregulated and 13 genes were downregulated. Overall, two genes (SPP1 and PLAU) were not only significantly upregulated in LUAD but also clearly related to poor prognosis (Figures 3A,B and Supplementary Figures 3A,B), which were selected as candidate hub genes. There were also four genes (AGTR1, OLR1, TEK, and TLR4) that were not only downregulated in LUAD but also significantly related to good survival (Figures 3C–F and Supplementary Figure 3C–F). Those genes were selected as qualified IRGs for further analysis.

### Immune Landscape of Hub Genes

**FIGURE 4:**
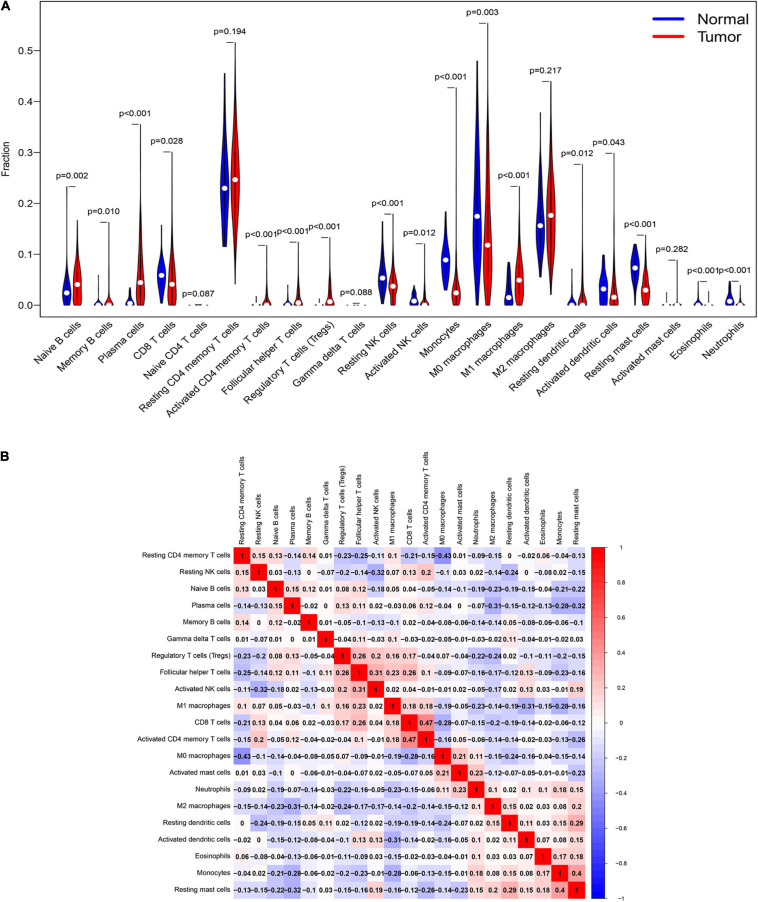
The immune signature of tumor infiltrating immune cells (TILs) in LUAD. **(A)** The proportions of TILs among different LUAD samples with a *P*-value < 0.05. **(B)** The correlation index among TILs in LUAD. The red color and blue color represent positive and negative correlation, respectively. The depth of color represents the degree of correlation index.

**FIGURE 5:**
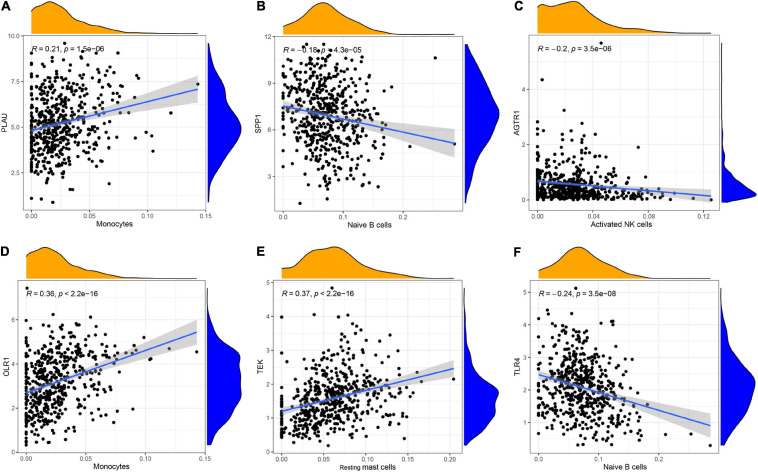
The relationship between IRGs and TILs in LUAD progression. **(A–C)** PLAU, OLR1, and TEK were the most strongly positively related to the proportions of monocytes (*R* = 0.21), monocytes (*R* = 0.36), and resting mast cells (*R* = 0.37), **(D–F)** whereas SPP1, AGTR1, and TLR4 were the most strongly negatively associated with the distributions of naive B cells (*R* = –0.18), activated NK cells (*R* = –0.2), and naive B cells (*R* = –0.24), respectively.

To understand the immune landscape of LUAD, we first evaluated the immune cells that exhibited significant changes in their proportions among the different samples, with a *P*-value < 0.05. As shown in Figure 4A, 17 types of cell showed significantly different distribution in LUAD. Among them, naive B cells, plasma cells, and M1 macrophages were significantly common in LUAD, whereas CD8 T cells, resting NK cells, activated NK cells, monocytes, M0 macrophages, activated dendritic cells, and resting mast cells showed significantly low rates in LUAD. In addition, the infiltration of resting CD4 memory T cells, M0 macrophages, and M2 macrophages showed relatively high levels compared with that of other immune cells (Figure 4A). We also assessed the correlations of the levels of these cells in LUAD. The results indicated that the distribution of monocytes was positively related to the proportion of resting mast cells, with a relation index (*R*) equal to 0.4, but the distribution of M0 macrophages was negatively related to the proportion of resting CD4 memory T cells, with *R* equal to −0.43 (Figure 4B). Furthermore, we estimated the correlation between qualified IRGs and immune cell, applying the following thresholds: *R* > 0.1 and *P*-value < 0.05. Overall, there were 3, 6, 4, 4, 6, and 7 types of immune cell related to AGTR1, OLR1, PLAU, SPP1, TEK, and TLR4 expression, respectively, according to our criteria. We present the highest relation index of each group in Figures 5A–F. PLAU, OLR1, and TEK were the most strongly positively related to the proportions of monocytes (*R* = 0.21), monocytes (*R* = 0.36), and resting mast cells (*R* = 0.37) separately, whereas SPP1, AGTR1, and TLR4 were the most strongly negatively associated with the distributions of naive B cells (*R* = −0.18), activated NK cells (*R* = −0.2), and naive B cells (*R* = −0.24), respectively.

### Constructing a ceRNA Network

**FIGURE 6:**
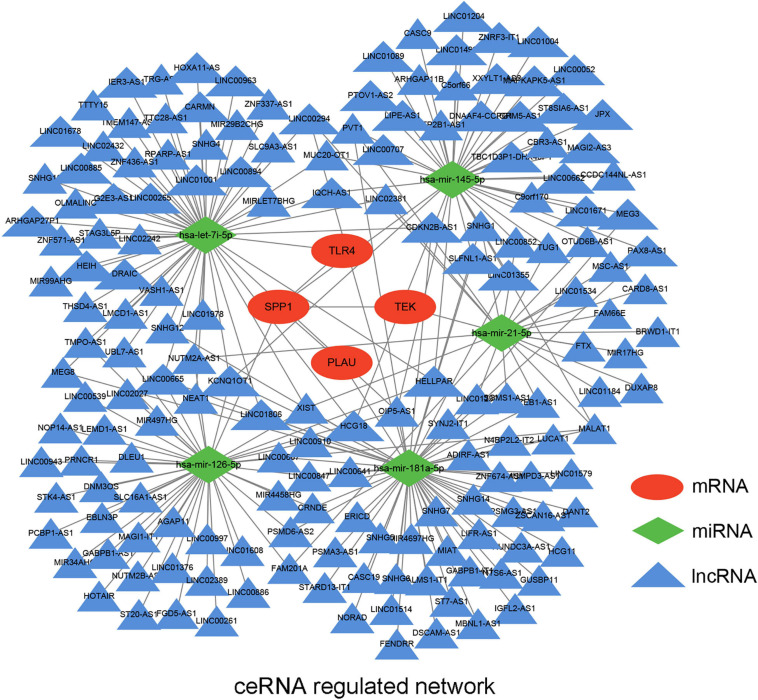
The potential mRNA-miRNA-lncRNA regulatory network related to LUAD immune signature. The ellipse, diamond, and triangle shape represent mRNA, miRNA, and lncRNA, respectively.

**FIGURE 7:**
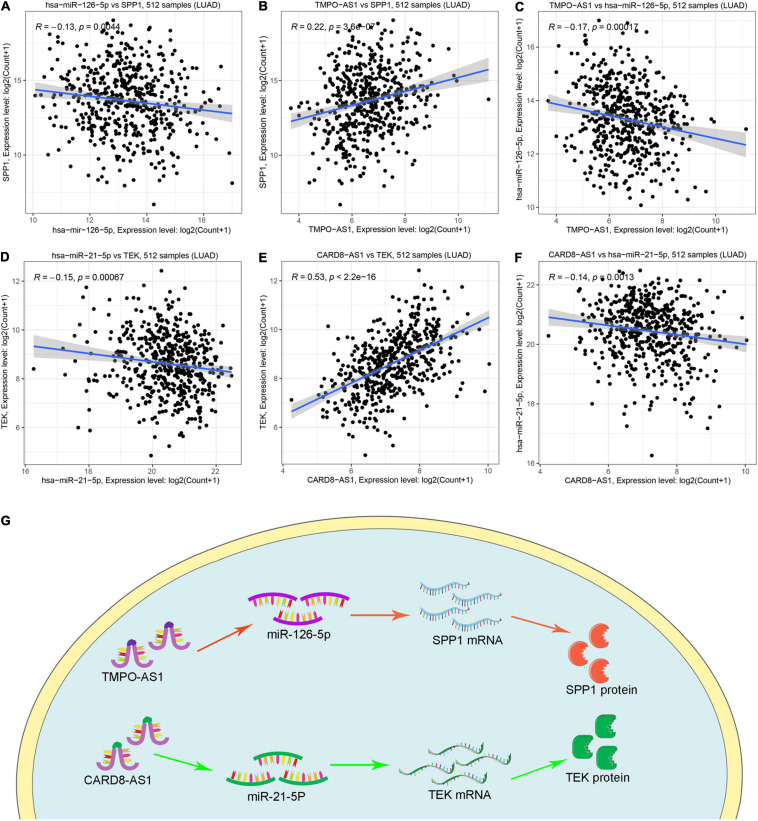
The co-expression analysis of ceRNA network and schematic representations. **(A–C)** The coexpression analysis indicated miR-126-5P was negatively correlated with SPP1 and TMPO-AS1, whereas TMPO-AS1 was positively correlated with SPP1. **(D–F)** The coexpression analysis indicated miR-21-5P was negatively correlated with TEK and CARD8-AS1, whereas CARD8-AS1 was positively correlated with TEK. **(G)** Schematic representations of TMPO-AS1/miR-126-5p/SPP1 and CARD8-AS1/miR-21-5p/TEK as immune-related ceRNA networks in LUAD progression.

Based on the previous identification and validation, six qualified IRGs were further predicted to obtain the upstream miRNAs through the TarBase. There were a total of 34 candidate miRNAs that were predicted to modulate the six qualified IRGs (Supplementary Table 2). Next, the starbase and KM plotter were applied to evaluate the expression levels and prognostic roles of the candidate miRNAs using the threshold of *P*-value < 0.05. As shown in Supplementary Figures 4, 5, we confirmed that five miRNAs interact with the qualified IRGs. Among them, two miRNAs (hsa-let-7i-5p, hsa-miR-21-5p) showed relatively high expression related to a dismal prognosis in LUAD, whereas three miRNAs showed low expression (hsa-miR-126-5p, hsa-miR-145-5p, and hsa-miR-181-5p) linked to prolonged survival. Additionally, we predicted the upstream lncRNAs of these five miRNAs using the miRNet database. A total of 224 lncRNAs were predicted to potentially interact with these five miRNAs (Supplementary Table 3). Furthermore, we constructed an lncRNA–miRNA–mRNA ceRNA network from our results. As presented in Figure 6, there were a total of 224 lncRNA–miRNA interactions and 5 miRNA–mRNA interactions in this network (Supplementary Table 4). Moreover, we evaluated the expression levels of the predicted lncRNAs in TCGA from our calculation and estimated their prognostic roles in the KM plotter database, applying a threshold of *P*-value < 0.05. Nine meaningful lncRNAs met our criteria (Supplementary Figure 6). Among them, six were highly expressed (HOTAIR, TMPO-AS1, LINC00707, IGFL2-AS1, LINC00665, and LUCAT1) related to a dismal prognosis, whereas three were expressed at a low level (CARD8-AS1, CARMN, and LINC00885) associated with prolonged survival. According to the ceRNA hypothesis, a qualified lncRNA should be negatively correlated with miRNA expression and positively correlated with the mRNA level at the same time. Therefore, we screened out all of the lncRNA–miRNA pairs and miRNA–mRNA pairs using co-expression analysis. Ultimately, we successfully validated two immune-related ceRNA networks (TMPO-AS1/miR-126-5p/SPP1 and CARD8-AS1/miR-21-5p/TEK) (Figures 7A–F) in LUAD progression through co-expression analysis. Also, we visualized these networks using schematic representations, as shown in Figure 7G. These networks could shed light on the oncogenesis of LUAD and may contain future diagnostic markers and therapeutic targets.

## Discussion

A malignant tumor is not merely an accumulation of neoplastic cells, but constitutes a microenvironment containing endothelial cells, fibroblasts, and infiltrating immune cells that impact on tumor development, invasion, metastasis, and outcome (Bremnes et al., 2016). Among these cells, TILs have been proven to be a major determinant of tumor characteristics and patient outcome (Stanton and Disis, 2016). However, their roles in LUAD remain to be elucidated. The ceRNA hypothesis has shed new light on the complex interaction between ncRNA and mRNA. In this hypothesis, ncRNAs act as “sponges” to absorb and bind miRNA, thereby weakening their ability to bind mRNA and regulating gene expression (Salmena et al., 2011). This mechanism is central to the progression and invasion of multiple types of cancer. For example, the NORAD/miR-125a-3p/RhoA ceRNA network facilitates the invasion and metastasis of pancreatic cancer (Li et al., 2017), the TRPM2-AS/miR-612/FOXM1 ceRNA network promotes GC cell progression, invasion, and radioresistance (Xiao et al., 2020), while the MALAT1/miR-200b/E2F3 ceRNA network induces docetaxel resistance in LUAD treatment (Chen J. et al., 2019). Therefore, it is essential to determine the interactions between IRGs and the ceRNA network in LUAD.

In the present study, we recognized six hub IRGs and evaluated their roles with TILs. We successfully confirmed the TMPO-AS1/miR-126-5p/SPP1 and CARD8-AS1/miR-21-5p/TEK networks as the immune-related ceRNA in LUAD progression. Studies have confirmed the roles of these six IRGs in cancer progression. For instance, the plasminogen activator (PLAU) can degrade the matrix surrounding a tumor and promote the migration of cells to distant organs, thereby inducing tumor cell invasion, migration, and homing to distant organs (Mahmood et al., 2018). Zeng et al. (2018) showed that SPP1 encouraged activation of the integrin β1/FAK/AKT pathway in SKOV3 and A2780 cells, which speeds up the cell proliferation, migration, and invasion of ovarian cancer. In addition, Turrini et al. (2017) identified a TIE-2/Tek receptor in tumor-associated macrophages and showed that its activator endowed these cells with pro-angiogenic activity, which could promote tumor invasion and metastasis and potentially be used as a biomarker of cancer progression. Notably, some ncRNAs also play pivotal roles in cancer progression. For example, miR-126-5p can reduce the enzymatic activity of MDH1 and mitochondrial respiration while sparing the function of its isoenzyme MDH2, which causes the death of NSCLC cells (Lima Queiroz et al., 2018). Also, as shown by Wang et al., miR-21-5p knockdown or WWC2 overexpression had an inhibitory effect on the proliferation, migration, and invasion of PC-9 cells in LUAD. However, such an effect was inhibited only when miR-21-5p was overexpressed, which indicated that miR-21-5p regulated WWC2 expression to facilitate LUAD progression (Wang G. et al., 2020). Moreover, Hu et al. (2020) discovered that TMPO-AS1 acted as a ceRNA for miR-126-5p to upregulate BRCC3 expression, and that BRCC3 was involved in the PI3K/Akt/mTOR pathway promoting the progression and invasion of gastric cancer. The luciferase reporter and RNA pull-down assay indicated that TMPO-AS1 could bind to miR-126-5p and serves as a ceRNA for miR-126-5p. Additionally, luciferase reporter indicated that miR-126-5p could inhibit SPP1 (OPN) expression by targeting SPP1 3’UTR sequences (Felli et al., 2013). Furthermore, in glioma, the silencing of CARD8-AS1 was shown to dramatically inhibit the proliferation and migration of U251 and A172 cells and induce their apoptosis (Lin et al., 2018). These studies indicated that our ceRNA network plays a crucial role in cancer progression and invasion, and has great potential as a therapeutic target for LUAD.

Additionally, the functional analysis indicated that the IRGs were also enriched in several immune processes such as immune response, inflammatory response, and cytokine activity. Those IRGs were also shown to be particularly associated with some immune-related pathways including cytokine receptor interaction pathway, HIF-1 signaling pathway, and TNF signaling pathway. In particular, Zhang et al. (2017) demonstrated that SPP1 facilitated macrophage polarization, which was upregulated by programmed death-ligand 1 and promoted immune escape, suggesting SPP1 as a potential therapeutic target for LUAD. In addition, as reported by He et al., PLAU could regulate the suppressor function of regulatory T cells (Tregs) via STAT5 and ERK signaling pathways. This function has been shown to be particularly important for memory Tregs (He et al., 2012). TLR4 is a number of Toll-like receptor (TLR) family, which plays a fundamental role in the activation of innate immunity. More importantly, Toll-like Receptor 4 (TLR4) agonists always have an application in the field of cancer immunotherapy (Shetab Boushehri and Lamprecht, 2018). This evidence confirms the close interactions between our IRG-related ceRNA and immune processes.

Although a few studies have already evaluated the roles of IRGs in cancer progression (Wan et al., 2019; Yang et al., 2019; Qiu et al., 2020), to our knowledge, this is the first study to detect the relationship between IRGs and the ceRNA network in LUAD. Our results are promising because our IRG-related ceRNA network showed important roles in the immune process. However, this study has some limitations. For instance, we mainly evaluated the top 20 hub IRGs in our ceRNA network, which might neglect the functions of other important IRGs. And we identified hub IRGs through the PPI network rather than other module identification methods, like FangNet, establishing a symptom-herb network using the weighted network and PageRank algorithm, which might neglect other hub genes in LUAD. However, their connective degree indicated their importance in the PPI network, so the top 20 hub IRGs should be representative and acceptable.

In addition, we confirmed the expression levels and prognostic roles using TCGA and GEO databases, rather than clinical samples, which could undermine the reliability of our work. It is for this reason that we constructed the ceRNA axis through integrative analysis and verified its importance in the different databases. Nonetheless, despite these limitations, we discovered and constructed two IRG-related ceRNA networks, which can be considered as an immunotherapeutic target for LUAD.

## Conclusion

In summary, by comprehensive analysis, we successfully constructed two ceRNA networks related to IRGs in LUAD progression. In our results, TMPO-AS1/miR-126-5p/SPP1 and CARD8-AS1/miR-21-5p/TEK are not only related to LUAD progression, but also function as immunotherapeutic targets for LUAD, clarifying a novel mechanism in cancer therapy.

## Data Availability Statement

The original contributions presented in the study are included in the article/Supplementary Material, further inquiries can be directed to the corresponding author/s.

## Author Contributions

YY, TZ, and KR conceived and designed this research. YY, TZ, JL, and KR performed data analysis and wrote the manuscript. All authors have read and approved the final manuscript.

## Conflict of Interest

The authors declare that the research was conducted in the absence of any commercial or financial relationships that could be construed as a potential conflict of interest.

## Supplementary Material

Supplementary Figure 1 Identifying immune-related genes (IRGs) in our cohort. **(A)** The IRGs among common DEGs and immune genes. **(B)** The protein-protein interaction (PPI) network of IRGs in the STRING database.

Supplementary Figure 2 Functional enrichment analysis for IRGs. **(A–C)** The top 10 enriched GO terms of IRGs with the threshold of *P*-value < 0.05 in biological processes (BP), cellular components (CC), and molecular functions (MF), respectively. **(D)** The top 10 enriched KEGG pathways of IRGs in the TCGA database.

Supplementary Figure 3 Validating the prognostic value of hub IRGs through OncoLnc database. **(A–F)** Validating prognostic roles of PLUA, SPP1, AGTR1, OLR1, TEK, and TLR4 through OncoLnc database, respectively (^∗^*P* < 0.05).

Supplementary Figure 4 Validating the expression level of candidate miRNAs in LUAD. **(A–E)** the expression level of hsa-miR-126-5p, hsa-miR-145-5p, hsa-miR-181-5p showed relatively low value in starbase, whereas hsa-let-7i-5p, hsa-miR-21-5p proved a relatively low expression.

Supplementary Figure 5 Validating the survival outcome of candidate miRNAs in LUAD. **(A–E)** hsa-miR-126-5p, hsa-miR-145-5p, hsa-miR-181-5p showed the prolonged survival, whereas hsa-let-7i-5p, hsa-miR-21-5p proved a dismal prognosis in LUAD.

Supplementary Figure 6 Screening and validating the expression level and prognostic value of meaningful lncRNAs in LUAD. **(A–I)** High expression of HOTAIR, TMPO-AS1, LINC00707, IGFL2-AS1, LINC00665, and LUCAT1 were related to a dismal prognosis, whereas low expression of CARD8-AS1, CARMN, and LINC00885 were associated with prolonged survival.

Supplementary Table 1 Differentially expressed genes (DEGs) and immune-related genes (IRGs) in our analysis.

Supplementary Table 2 The mRNA-miRNA pairs predicted by the TarBase database.

Supplementary Table 3 The miRNA-lncRNA pairs predicted by the miRNet database.

Supplementary Table 4 The qualified lncRNA-miRNA and miRNA-mRNA pairs were constructed in the ceRNA network.
