# Supplementary figures and images for: Identification of a Competing Endogenous RNA Network Related to Immune Signature in Lung Adenocarcinoma
Source: Front Genet. 2021 Jun 3;12:665555. doi: 10.3389/fgene.2021.665555 (PMC8209499; doi:10.3389/fgene.2021.665555)

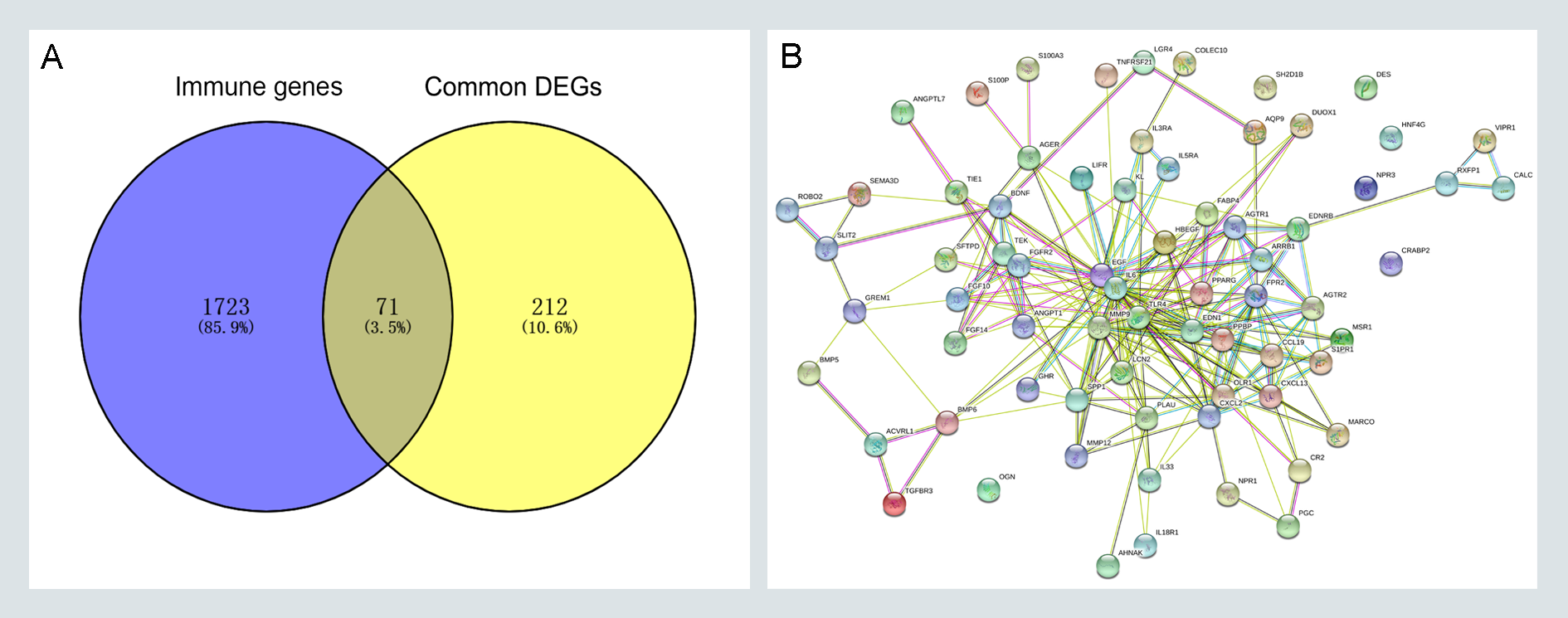

Supplement: Supplementary Figure 1 — Identifying immune-related genes (IRGs) in our cohort. (A) The IRGs among common DEGs and immune genes. (B) The protein-protein interaction (PPI) network of IRGs in the STRING database. [file Image_1.TIF]

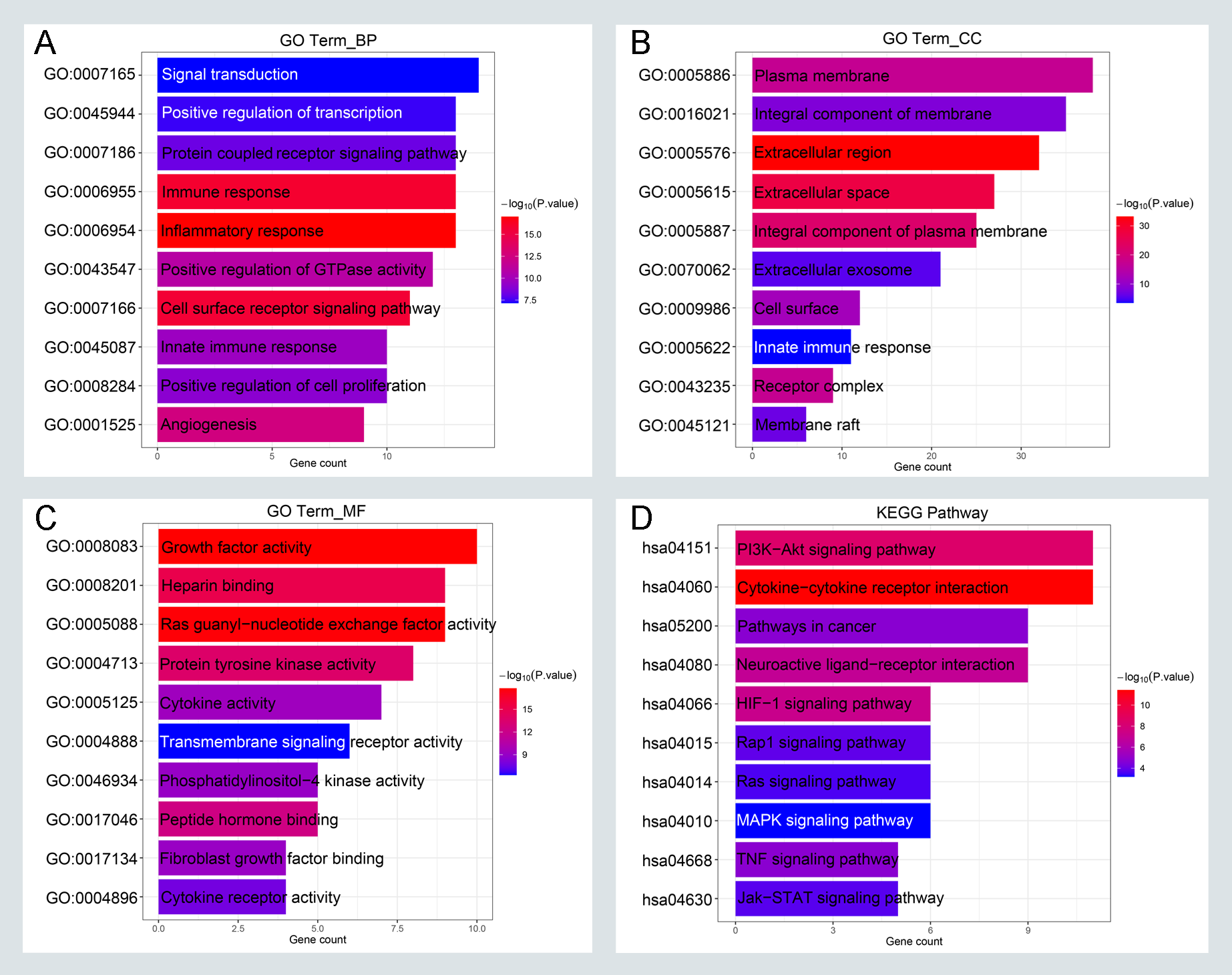

Supplement: Supplementary Figure 2 — Functional enrichment analysis for IRGs. (A–C) The top 10 enriched GO terms of IRGs with the threshold of P-value < 0.05 in biological processes (BP), cellular components (CC), and molecular functions (MF), respectively. (D) The top 10 enriched KEGG pathways of IRGs in the TCGA database. [file Image_2.TIF]

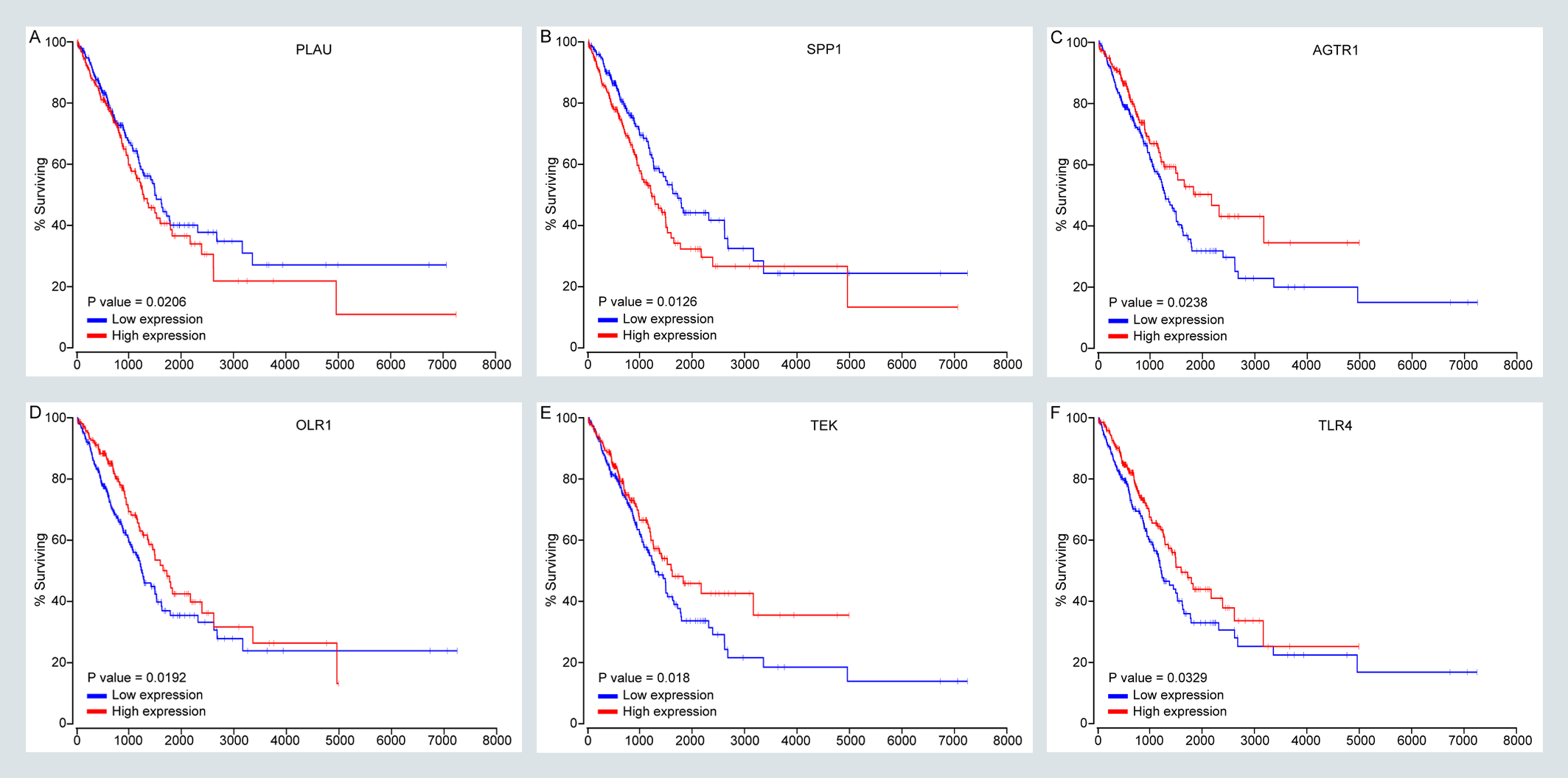

Supplement: Supplementary Figure 3 — Validating the prognostic value of hub IRGs through OncoLnc database. (A–F) Validating prognostic roles of PLUA, SPP1, AGTR1, OLR1, TEK, and TLR4 through OncoLnc database, respectively (∗P < 0.05). [file Image_3.TIF]

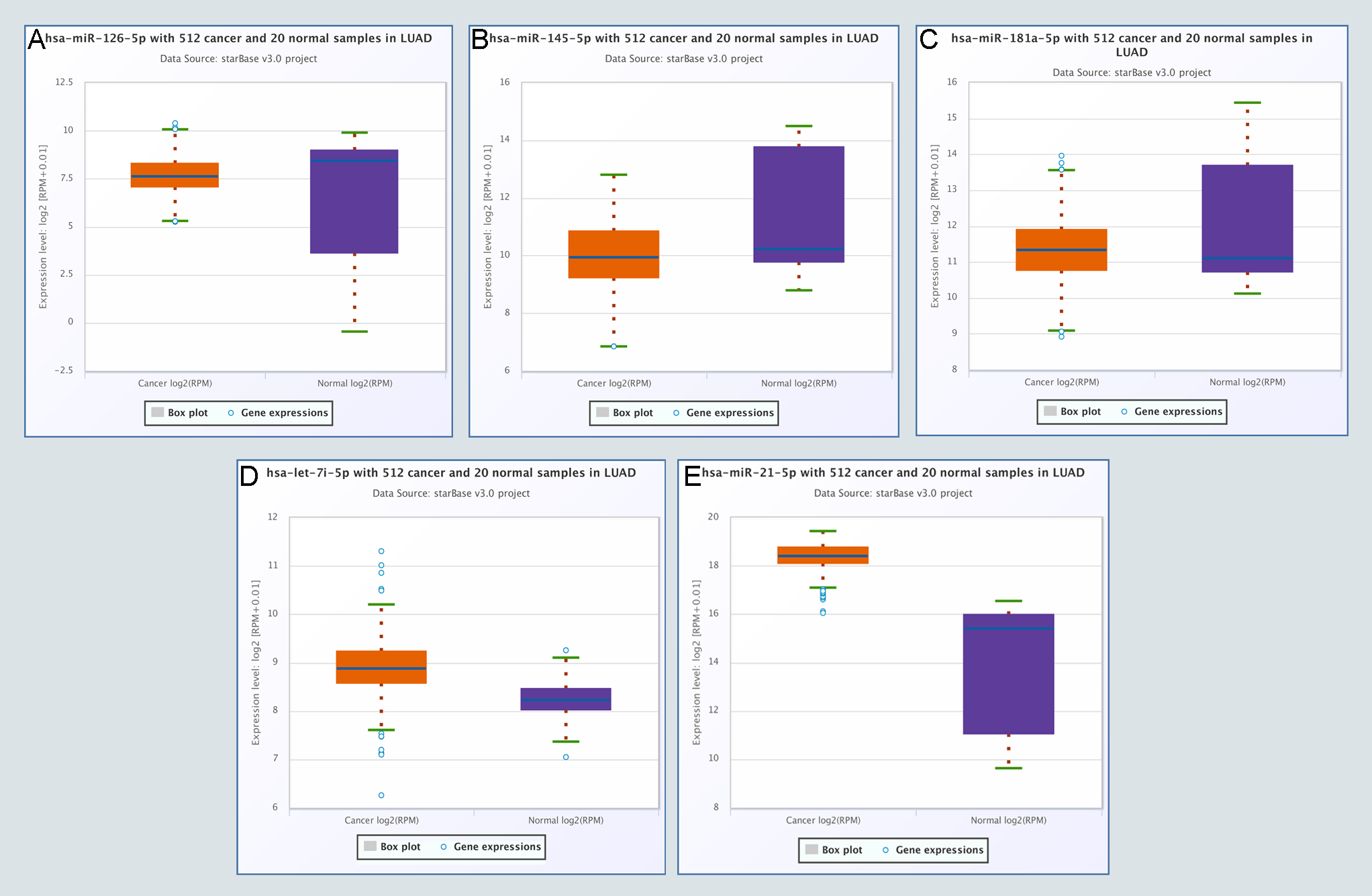

Supplement: Supplementary Figure 4 — Validating the expression level of candidate miRNAs in LUAD. (A–E) the expression level of hsa-miR-126-5p, hsa-miR-145-5p, hsa-miR-181-5p showed relatively low value in starbase, whereas hsa-let-7i-5p, hsa-miR-21-5p proved a relatively low expression. [file Image_4.TIF]

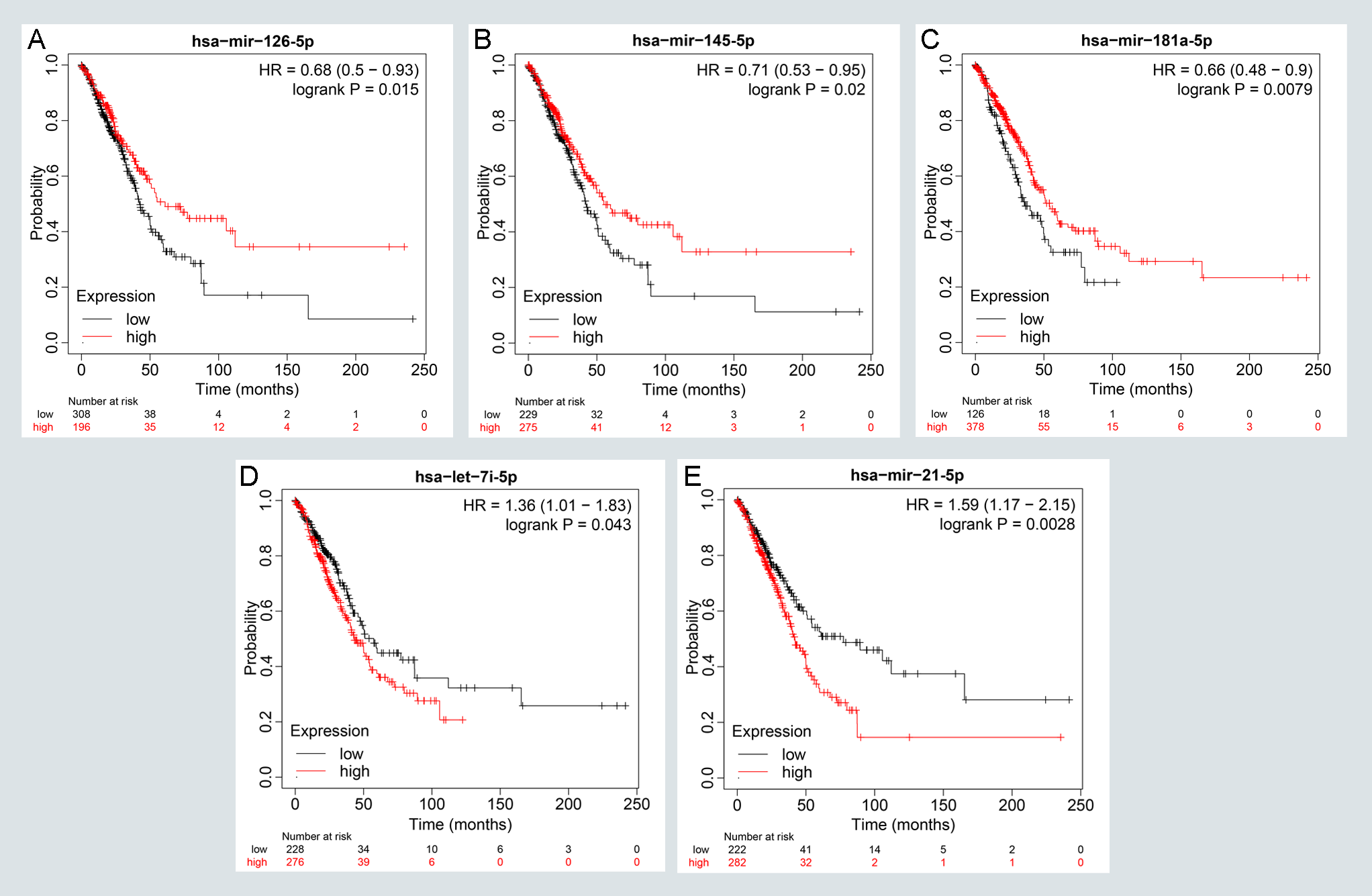

Supplement: Supplementary Figure 5 — Validating the survival outcome of candidate miRNAs in LUAD. (A–E) hsa-miR-126-5p, hsa-miR-145-5p, hsa-miR-181-5p showed the prolonged survival, whereas hsa-let-7i-5p, hsa-miR-21-5p proved a dismal prognosis in LUAD. [file Image_5.TIF]

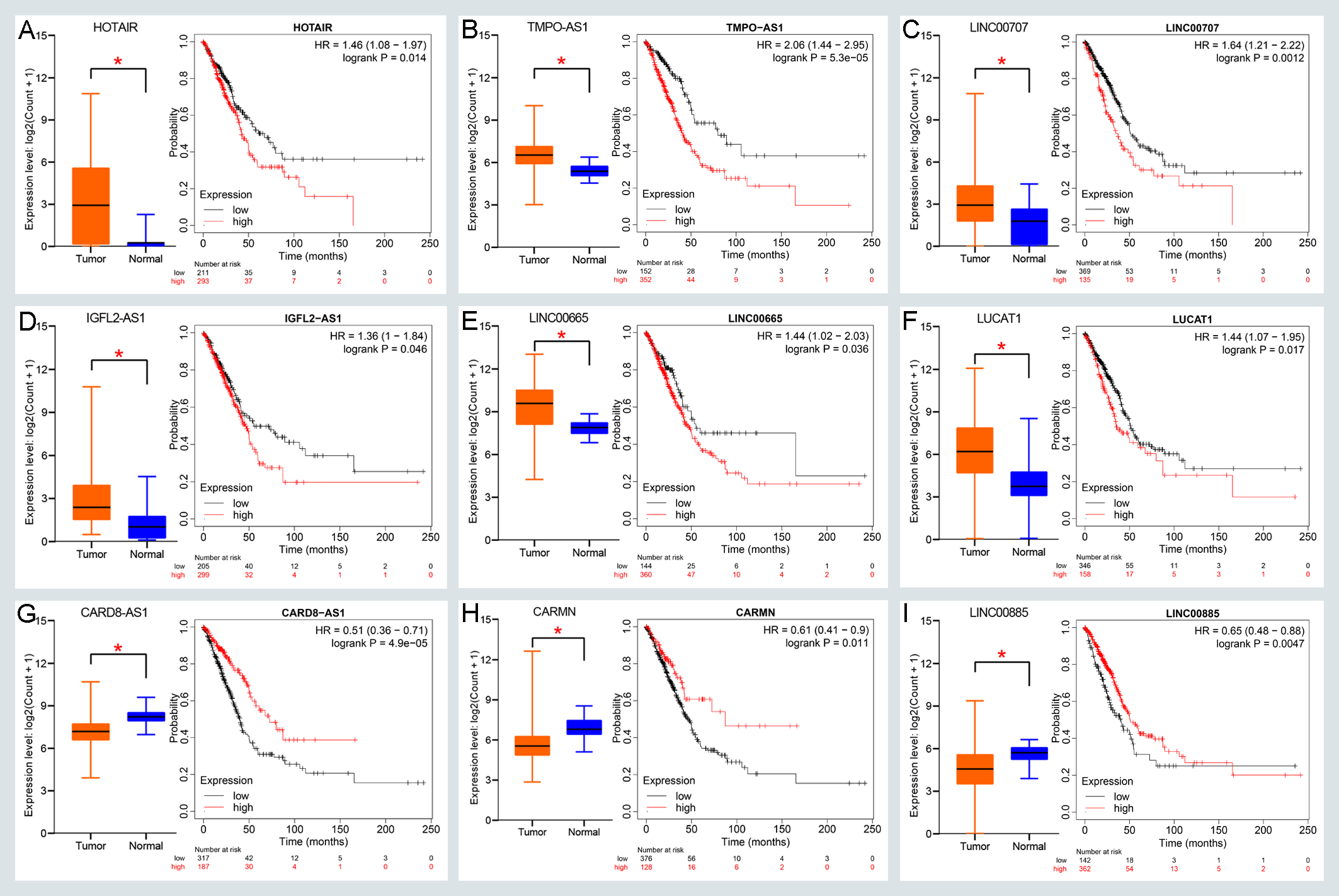

Supplement: Supplementary Figure 6 — Screening and validating the expression level and prognostic value of meaningful lncRNAs in LUAD. (A–I) High expression of HOTAIR, TMPO-AS1, LINC00707, IGFL2-AS1, LINC00665, and LUCAT1 were related to a dismal prognosis, whereas low expression of CARD8-AS1, CARMN, and LINC00885 were associated with prolonged survival. [file Image_6.TIF]
