# Supplementary material for: Identification of a Competing Endogenous RNA Network Related to Immune Signature in Lung Adenocarcinoma
Source: Front Genet. 2021 Jun 3;12:665555. doi: 10.3389/fgene.2021.665555 (PMC8209499; doi:10.3389/fgene.2021.665555)
Supplement: Supplementary Table 2 — The mRNA-miRNA pairs predicted by the TarBase database. [file Table_2.DOCX]

Supplementary Table 2. The mRNA-miRNA pairs predicted by the TarBase database.

| mRNA | miRNA |
| --- | --- |
| TLR4 | hsa-miR-146a-5p |
| TLR4 | hsa-let-7b-5p |
| TLR4 | hsa-miR-26b-5p |
| TLR4 | hsa-miR-146b-5p |
| TLR4 | hsa-let-7i-5p |
| TLR4 | hsa-miR-181b-3p |
| TLR4 | hsa-let-7i |
| SPP1 | hsa-miR-299-5p |
| SPP1 | hsa-miR-126-5p |
| SPP1 | hsa-miR-126-3p |
| SPP1 | hsa-miR-181b-5p |
| SPP1 | hsa-miR-4262 |
| SPP1 | hsa-miR-127-5p |
| SPP1 | hsa-miR-145-5p |
| SPP1 | hsa-miR-27a-5p |
| SPP1 | hsa-miR-146a-5p |
| AGTR1 | hsa-miR-155-5p |
| AGTR1 | hsa-miR-34a-5p |
| AGTR1 | hsa-miR-410-3p |
| TEK | hsa-miR-21-5p |
| TEK | hsa-miR-126-3p |
| OLR1 | hsa-miR-155-5p |
| OLR1 | hsa-miR-590-5p |
| PLAU | hsa-miR-193a-3p |
| PLAU | hsa-miR-193b-3p |
| PLAU | hsa-miR-181a-5p |
| PLAU | hsa-miR-23b-3p |
| PLAU | hsa-miR-195-5p |
| PLAU | hsa-miR-34a-5p |
| PLAU | hsa-miR-128-3p |
| PLAU | hsa-miR-143-3p |
| PLAU | hsa-miR-494-3p |
| PLAU | hsa-miR-23b |
| PLAU | hsa-miR-193b |
