# Supplementary material for: Identification of a Competing Endogenous RNA Network Related to Immune Signature in Lung Adenocarcinoma
Source: Front Genet. 2021 Jun 3;12:665555. doi: 10.3389/fgene.2021.665555 (PMC8209499; doi:10.3389/fgene.2021.665555)
Supplement: Supplementary Table 3 — The miRNA-lncRNA pairs predicted by the miRNet database. [file Table_3.DOCX]

Supplementary Table 3. The miRNA-lncRNA pairs predicted by the miRNet database.

| miRNA | lncRNA |
| --- | --- |
| hsa-miR-126-5p | AGAP11 |
| hsa-miR-126-5p | CRNDE |
| hsa-miR-126-5p | DLEU1 |
| hsa-miR-126-5p | DNM3OS |
| hsa-miR-126-5p | EBLN3P |
| hsa-miR-126-5p | FAM201A |
| hsa-miR-126-5p | FGD5-AS1 |
| hsa-miR-126-5p | GABPB1-AS1 |
| hsa-miR-126-5p | HELLPAR |
| hsa-miR-126-5p | HOTAIR |
| hsa-miR-126-5p | KCNQ1OT1 |
| hsa-miR-126-5p | LEMD1-AS1 |
| hsa-miR-126-5p | LINC00261 |
| hsa-miR-126-5p | LINC00539 |
| hsa-miR-126-5p | LINC00665 |
| hsa-miR-126-5p | LINC00667 |
| hsa-miR-126-5p | LINC00847 |
| hsa-miR-126-5p | LINC00886 |
| hsa-miR-126-5p | LINC00910 |
| hsa-miR-126-5p | LINC00943 |
| hsa-miR-126-5p | RSF1-IT1 |
| hsa-miR-126-5p | SGMS1-AS1 |
| hsa-miR-126-5p | LINC00997 |
| hsa-miR-126-5p | LINC01376 |
| hsa-miR-126-5p | LINC01608 |
| hsa-miR-126-5p | LINC02027 |
| hsa-miR-126-5p | LINC02389 |
| hsa-miR-126-5p | MAGI1-IT1 |
| hsa-miR-126-5p | MALAT1 |
| hsa-miR-126-5p | MIR34AHG |
| hsa-miR-126-5p | MIR4458HG |
| hsa-miR-126-5p | MIR497HG |
| hsa-miR-126-5p | NEAT1 |
| hsa-miR-126-5p | NOP14-AS1 |
| hsa-miR-126-5p | NUTM2A-AS1 |
| hsa-miR-126-5p | NUTM2B-AS1 |
| hsa-miR-126-5p | OIP5-AS1 |
| hsa-miR-126-5p | PCBP1-AS1 |
| hsa-miR-126-5p | PRNCR1 |
| hsa-miR-126-5p | PSMD6-AS2 |
| hsa-miR-126-5p | SLC16A1-AS1 |
| hsa-miR-126-5p | ST20-AS1 |
| hsa-miR-126-5p | STK4-AS1 |
| hsa-miR-126-5p | TMPO-AS1 |
| hsa-miR-126-5p | UBL7-AS1 |
| hsa-miR-126-5p | XIST |
| hsa-miR-145-5p | ARHGAP11B |
| hsa-miR-145-5p | ATP2B1-AS1 |
| hsa-miR-145-5p | C5orf66 |
| hsa-miR-145-5p | C9orf170 |
| hsa-miR-145-5p | CASC9 |
| hsa-miR-145-5p | CBR3-AS1 |
| hsa-miR-145-5p | CCDC144NL-AS1 |
| hsa-miR-145-5p | DNAAF4-CCPG1 |
| hsa-miR-145-5p | GRM5-AS1 |
| hsa-miR-145-5p | HCG18 |
| hsa-miR-145-5p | HELLPAR |
| hsa-miR-145-5p | IQCH-AS1 |
| hsa-miR-145-5p | JPX |
| hsa-miR-145-5p | KCNQ1OT1 |
| hsa-miR-145-5p | LINC00052 |
| hsa-miR-145-5p | LINC00662 |
| hsa-miR-145-5p | LINC00707 |
| hsa-miR-145-5p | LINC00852 |
| hsa-miR-145-5p | LINC01004 |
| hsa-miR-145-5p | LINC01089 |
| hsa-miR-145-5p | LINC01204 |
| hsa-miR-145-5p | LINC01355 |
| hsa-miR-145-5p | LINC01499 |
| hsa-miR-145-5p | LINC01671 |
| hsa-miR-145-5p | LIPE-AS1 |
| hsa-miR-145-5p | MAGI2-AS3 |
| hsa-miR-145-5p | MALAT1 |
| hsa-miR-145-5p | MAPKAPK5-AS1 |
| hsa-miR-145-5p | MEG3 |
| hsa-miR-145-5p | MUC20-OT1 |
| hsa-miR-145-5p | OIP5-AS1 |
| hsa-miR-145-5p | OTUD6B-AS1 |
| hsa-miR-145-5p | PAX8-AS1 |
| hsa-miR-145-5p | PTOV1-AS2 |
| hsa-miR-145-5p | PVT1 |
| hsa-miR-145-5p | SLFNL1-AS1 |
| hsa-miR-145-5p | SNHG1 |
| hsa-miR-145-5p | ST8SIA6-AS1 |
| hsa-miR-145-5p | TBC1D3P1-DHX40P1 |
| hsa-miR-145-5p | TUG1 |
| hsa-miR-145-5p | XXYLT1-AS2 |
| hsa-miR-145-5p | ZNRF3-IT1 |
| hsa-miR-181a-5p | ADIRF-AS1 |
| hsa-miR-181a-5p | ALMS1-IT1 |
| hsa-miR-181a-5p | CASC19 |
| hsa-miR-181a-5p | CDKN2B-AS1 |
| hsa-miR-181a-5p | CRNDE |
| hsa-miR-181a-5p | DANT2 |
| hsa-miR-181a-5p | DSCAM-AS1 |
| hsa-miR-181a-5p | ERICD |
| hsa-miR-181a-5p | FENDRR |
| hsa-miR-181a-5p | GABPB1-IT1 |
| hsa-miR-181a-5p | GUSBP11 |
| hsa-miR-181a-5p | HCG11 |
| hsa-miR-181a-5p | HELLPAR |
| hsa-miR-181a-5p | IGFL2-AS1 |
| hsa-miR-181a-5p | INTS6-AS1 |
| hsa-miR-181a-5p | KCNQ1OT1 |
| hsa-miR-181a-5p | LIFR-AS1 |
| hsa-miR-181a-5p | LINC00294 |
| hsa-miR-181a-5p | LINC00641 |
| hsa-miR-181a-5p | LINC00665 |
| hsa-miR-181a-5p | LINC00667 |
| hsa-miR-181a-5p | LINC00847 |
| hsa-miR-181a-5p | LINC00910 |
| hsa-miR-181a-5p | LINC01232 |
| hsa-miR-181a-5p | LINC01355 |
| hsa-miR-181a-5p | LINC01514 |
| hsa-miR-181a-5p | LINC01579 |
| hsa-miR-181a-5p | LINC01806 |
| hsa-miR-181a-5p | LUCAT1 |
| hsa-miR-181a-5p | MALAT1 |
| hsa-miR-181a-5p | MBNL1-AS1 |
| hsa-miR-181a-5p | MEG3 |
| hsa-miR-181a-5p | MEG8 |
| hsa-miR-181a-5p | MIAT |
| hsa-miR-181a-5p | MIR4458HG |
| hsa-miR-181a-5p | MIR4697HG |
| hsa-miR-181a-5p | N4BP2L2-IT2 |
| hsa-miR-181a-5p | NEAT1 |
| hsa-miR-181a-5p | NORAD |
| hsa-miR-181a-5p | OIP5-AS1 |
| hsa-miR-181a-5p | PAX8-AS1 |
| hsa-miR-181a-5p | PSMA3-AS1 |
| hsa-miR-181a-5p | PSMD6-AS2 |
| hsa-miR-181a-5p | PSMG3-AS1 |
| hsa-miR-181a-5p | PVT1 |
| hsa-miR-181a-5p | RUNDC3A-AS1 |
| hsa-miR-181a-5p | SGMS1-AS1 |
| hsa-miR-181a-5p | SLFNL1-AS1 |
| hsa-miR-181a-5p | SNHG1 |
| hsa-miR-181a-5p | SNHG12 |
| hsa-miR-181a-5p | SNHG14 |
| hsa-miR-181a-5p | SNHG5 |
| hsa-miR-181a-5p | SNHG6 |
| hsa-miR-181a-5p | SNHG7 |
| hsa-miR-181a-5p | ST7-AS1 |
| hsa-miR-181a-5p | STARD13-IT1 |
| hsa-miR-181a-5p | SYNJ2-IT1 |
| hsa-miR-181a-5p | THUMPD3-AS1 |
| hsa-miR-181a-5p | XIST |
| hsa-miR-181a-5p | ZEB1-AS1 |
| hsa-miR-181a-5p | ZNF674-AS1 |
| hsa-miR-181a-5p | ZSCAN16-AS1 |
| hsa-miR-21-5p | BRWD1-IT1 |
| hsa-miR-21-5p | CARD8-AS1 |
| hsa-miR-21-5p | DUXAP8 |
| hsa-miR-21-5p | FAM201A |
| hsa-miR-21-5p | FAM66E |
| hsa-miR-21-5p | FTX |
| hsa-miR-21-5p | LINC00294 |
| hsa-miR-21-5p | LINC00852 |
| hsa-miR-21-5p | LINC01184 |
| hsa-miR-21-5p | LINC01232 |
| hsa-miR-21-5p | LINC01534 |
| hsa-miR-21-5p | LINC02381 |
| hsa-miR-21-5p | MALAT1 |
| hsa-miR-21-5p | MIR17HG |
| hsa-miR-21-5p | MSC-AS1 |
| hsa-miR-21-5p | NUTM2A-AS1 |
| hsa-miR-21-5p | OTUD6B-AS1 |
| hsa-miR-21-5p | PVT1 |
| hsa-miR-21-5p | SGMS1-AS1 |
| hsa-miR-21-5p | SNHG1 |
| hsa-miR-21-5p | TUG1 |
| hsa-miR-21-5p | XIST |
| hsa-miR-21-5p | ZEB1-AS1 |
| hsa-let-7i-5p | ARHGAP27P1 |
| hsa-let-7i-5p | CARMN |
| hsa-let-7i-5p | CDKN2B-AS1 |
| hsa-let-7i-5p | DRAIC |
| hsa-let-7i-5p | G2E3-AS1 |
| hsa-let-7i-5p | HCG18 |
| hsa-let-7i-5p | HEIH |
| hsa-let-7i-5p | HELLPAR |
| hsa-let-7i-5p | HOXA11-AS |
| hsa-let-7i-5p | IER3-AS1 |
| hsa-let-7i-5p | IQCH-AS1 |
| hsa-let-7i-5p | KCNQ1OT1 |
| hsa-let-7i-5p | LINC00265 |
| hsa-let-7i-5p | LINC00294 |
| hsa-let-7i-5p | LINC00665 |
| hsa-let-7i-5p | LINC00885 |
| hsa-let-7i-5p | LINC00894 |
| hsa-let-7i-5p | LINC00963 |
| hsa-let-7i-5p | LINC01001 |
| hsa-let-7i-5p | LINC01678 |
| hsa-let-7i-5p | LINC01806 |
| hsa-let-7i-5p | LINC01978 |
| hsa-let-7i-5p | LINC02242 |
| hsa-let-7i-5p | LINC02381 |
| hsa-let-7i-5p | LINC02432 |
| hsa-let-7i-5p | LMCD1-AS1 |
| hsa-let-7i-5p | MEG8 |
| hsa-let-7i-5p | MIR29B2CHG |
| hsa-let-7i-5p | MIR99AHG |
| hsa-let-7i-5p | MIRLET7BHG |
| hsa-let-7i-5p | MUC20-OT1 |
| hsa-let-7i-5p | NEAT1 |
| hsa-let-7i-5p | NUTM2A-AS1 |
| hsa-let-7i-5p | OIP5-AS1 |
| hsa-let-7i-5p | OLMALINC |
| hsa-let-7i-5p | RPARP-AS1 |
| hsa-let-7i-5p | SLC9A3-AS1 |
| hsa-let-7i-5p | SNHG12 |
| hsa-let-7i-5p | SNHG16 |
| hsa-let-7i-5p | SNHG4 |
| hsa-let-7i-5p | STAG3L5P |
| hsa-let-7i-5p | THSD4-AS1 |
| hsa-let-7i-5p | TMEM147-AS1 |
| hsa-let-7i-5p | TMPO-AS1 |
| hsa-let-7i-5p | TRG-AS1 |
| hsa-let-7i-5p | TTC28-AS1 |
| hsa-let-7i-5p | TTTY15 |
| hsa-let-7i-5p | UBL7-AS1 |
| hsa-let-7i-5p | VASH1-AS1 |
| hsa-let-7i-5p | XIST |
| hsa-let-7i-5p | ZNF337-AS1 |
| hsa-let-7i-5p | ZNF436-AS1 |
| hsa-let-7i-5p | ZNF571-AS1 |
