# Supplementary material for: Identification of a Competing Endogenous RNA Network Related to Immune Signature in Lung Adenocarcinoma
Source: Front Genet. 2021 Jun 3;12:665555. doi: 10.3389/fgene.2021.665555 (PMC8209499; doi:10.3389/fgene.2021.665555)
Supplement: Supplementary Table 4 — The qualified lncRNA-miRNA and miRNA-mRNA pairs were constructed in the ceRNA network. [file Table_4.DOCX]

**Supplementary Table 4**. The qualified lncRNA-miRNA and miRNA-mRNA pairs were constructed in the ceRNA network.

| Node1 | Node2 | Type |
| --- | --- | --- |
| SPP1 | OLR1 | mRNA |
| SPP1 | PLAU | mRNA |
| SPP1 | TEK | mRNA |
| SPP1 | TLR4 | mRNA |
| TLR4 | hsa-let-7i-5p | mRNA |
| TEK | hsa-miR-21-5p | mRNA |
| SPP1 | hsa-miR-126-5p | mRNA |
| SPP1 | hsa-miR-145-5p | mRNA |
| PLAU | hsa-miR-181a-5p | mRNA |
| hsa-let-7i-5p | ARHGAP27P1 | lncRNA |
| hsa-let-7i-5p | CARMN | lncRNA |
| hsa-let-7i-5p | CDKN2B-AS1 | lncRNA |
| hsa-let-7i-5p | DRAIC | lncRNA |
| hsa-let-7i-5p | G2E3-AS1 | lncRNA |
| hsa-let-7i-5p | HCG18 | lncRNA |
| hsa-let-7i-5p | HEIH | lncRNA |
| hsa-let-7i-5p | HELLPAR | lncRNA |
| hsa-let-7i-5p | HOXA11-AS | lncRNA |
| hsa-let-7i-5p | IER3-AS1 | lncRNA |
| hsa-let-7i-5p | IQCH-AS1 | lncRNA |
| hsa-let-7i-5p | KCNQ1OT1 | lncRNA |
| hsa-let-7i-5p | LINC00265 | lncRNA |
| hsa-let-7i-5p | LINC00294 | lncRNA |
| hsa-let-7i-5p | LINC00665 | lncRNA |
| hsa-let-7i-5p | LINC00885 | lncRNA |
| hsa-let-7i-5p | LINC00894 | lncRNA |
| hsa-let-7i-5p | LINC00963 | lncRNA |
| hsa-let-7i-5p | LINC01001 | lncRNA |
| hsa-let-7i-5p | LINC01678 | lncRNA |
| hsa-let-7i-5p | LINC01806 | lncRNA |
| hsa-let-7i-5p | LINC01978 | lncRNA |
| hsa-let-7i-5p | LINC02242 | lncRNA |
| hsa-let-7i-5p | LINC02381 | lncRNA |
| hsa-let-7i-5p | LINC02432 | lncRNA |
| hsa-let-7i-5p | LMCD1-AS1 | lncRNA |
| hsa-let-7i-5p | MEG8 | lncRNA |
| hsa-let-7i-5p | MIR29B2CHG | lncRNA |
| hsa-let-7i-5p | MIR99AHG | lncRNA |
| hsa-let-7i-5p | MIRLET7BHG | lncRNA |
| hsa-let-7i-5p | MUC20-OT1 | lncRNA |
| hsa-let-7i-5p | NEAT1 | lncRNA |
| hsa-let-7i-5p | NUTM2A-AS1 | lncRNA |
| hsa-let-7i-5p | OIP5-AS1 | lncRNA |
| hsa-let-7i-5p | OLMALINC | lncRNA |
| hsa-let-7i-5p | RPARP-AS1 | lncRNA |
| hsa-let-7i-5p | SLC9A3-AS1 | lncRNA |
| hsa-let-7i-5p | SNHG12 | lncRNA |
| hsa-let-7i-5p | SNHG16 | lncRNA |
| hsa-let-7i-5p | SNHG4 | lncRNA |
| hsa-let-7i-5p | STAG3L5P | lncRNA |
| hsa-let-7i-5p | THSD4-AS1 | lncRNA |
| hsa-let-7i-5p | TMEM147-AS1 | lncRNA |
| hsa-let-7i-5p | TMPO-AS1 | lncRNA |
| hsa-let-7i-5p | TRG-AS1 | lncRNA |
| hsa-let-7i-5p | TTC28-AS1 | lncRNA |
| hsa-let-7i-5p | TTTY15 | lncRNA |
| hsa-let-7i-5p | UBL7-AS1 | lncRNA |
| hsa-let-7i-5p | VASH1-AS1 | lncRNA |
| hsa-let-7i-5p | XIST | lncRNA |
| hsa-let-7i-5p | ZNF337-AS1 | lncRNA |
| hsa-let-7i-5p | ZNF436-AS1 | lncRNA |
| hsa-let-7i-5p | ZNF571-AS1 | lncRNA |
| hsa-miR-21-5p | BRWD1-IT1 | lncRNA |
| hsa-miR-21-5p | CARD8-AS1 | lncRNA |
| hsa-miR-21-5p | DUXAP8 | lncRNA |
| hsa-miR-21-5p | FAM201A | lncRNA |
| hsa-miR-21-5p | FAM66E | lncRNA |
| hsa-miR-21-5p | FTX | lncRNA |
| hsa-miR-21-5p | LINC00294 | lncRNA |
| hsa-miR-21-5p | LINC00852 | lncRNA |
| hsa-miR-21-5p | LINC01184 | lncRNA |
| hsa-miR-21-5p | LINC01232 | lncRNA |
| hsa-miR-21-5p | LINC01534 | lncRNA |
| hsa-miR-21-5p | LINC02381 | lncRNA |
| hsa-miR-21-5p | MALAT | lncRNA |
| hsa-miR-21-5p | MIR17HG | lncRNA |
| hsa-miR-21-5p | MSC-AS1 | lncRNA |
| hsa-miR-21-5p | NUTM2A-AS1 | lncRNA |
| hsa-miR-21-5p | OTUD6B-AS1 | lncRNA |
| hsa-miR-21-5p | PVT1 | lncRNA |
| hsa-miR-21-5p | SGMS1-AS1 | lncRNA |
| hsa-miR-21-5p | SNHG1 | lncRNA |
| hsa-miR-21-5p | TUG1 | lncRNA |
| hsa-miR-21-5p | XIST | lncRNA |
| hsa-miR-21-5p | ZEB1-AS1 | lncRNA |
| hsa-miR-126-5p | AGAP11 | lncRNA |
| hsa-miR-126-5p | CRNDE | lncRNA |
| hsa-miR-126-5p | DLEU1 | lncRNA |
| hsa-miR-126-5p | DNM3OS | lncRNA |
| hsa-miR-126-5p | EBLN3P | lncRNA |
| hsa-miR-126-5p | FAM201A | lncRNA |
| hsa-miR-126-5p | FGD5-AS1 | lncRNA |
| hsa-miR-126-5p | GABPB1-AS1 | lncRNA |
| hsa-miR-126-5p | HELLPAR | lncRNA |
| hsa-miR-126-5p | HOTAIR | lncRNA |
| hsa-miR-126-5p | KCNQ1OT1 | lncRNA |
| hsa-miR-126-5p | LEMD1-AS1 | lncRNA |
| hsa-miR-126-5p | LINC00261 | lncRNA |
| hsa-miR-126-5p | LINC00539 | lncRNA |
| hsa-miR-126-5p | LINC00665 | lncRNA |
| hsa-miR-126-5p | LINC00667 | lncRNA |
| hsa-miR-126-5p | LINC00847 | lncRNA |
| hsa-miR-126-5p | LINC00886 | lncRNA |
| hsa-miR-126-5p | LINC00910 | lncRNA |
| hsa-miR-126-5p | LINC00943 | lncRNA |
| hsa-miR-126-5p | LINC00997 | lncRNA |
| hsa-miR-126-5p | LINC01376 | lncRNA |
| hsa-miR-126-5p | LINC01608 | lncRNA |
| hsa-miR-126-5p | LINC02027 | lncRNA |
| hsa-miR-126-5p | LINC02389 | lncRNA |
| hsa-miR-126-5p | MAGI1-IT1 | lncRNA |
| hsa-miR-126-5p | MALAT1 | lncRNA |
| hsa-miR-126-5p | MIR34AHG | lncRNA |
| hsa-miR-126-5p | MIR4458HG | lncRNA |
| hsa-miR-126-5p | MIR497HG | lncRNA |
| hsa-miR-126-5p | NEAT1 | lncRNA |
| hsa-miR-126-5p | NOP14-AS1 | lncRNA |
| hsa-miR-126-5p | NUTM2A-AS1 | lncRNA |
| hsa-miR-126-5p | NUTM2B-AS1 | lncRNA |
| hsa-miR-126-5p | OIP5-AS1 | lncRNA |
| hsa-miR-126-5p | PCBP1-AS1 | lncRNA |
| hsa-miR-126-5p | PRNCR1 | lncRNA |
| hsa-miR-126-5p | PSMD6-AS2 | lncRNA |
| hsa-miR-126-5p | SLC16A1-AS1 | lncRNA |
| hsa-miR-126-5p | ST20-AS1 | lncRNA |
| hsa-miR-126-5p | STK4-AS1 | lncRNA |
| hsa-miR-126-5p | TMPO-AS1 | lncRNA |
| hsa-miR-126-5p | UBL7-AS1 | lncRNA |
| hsa-miR-126-5p | XIST | lncRNA |
| hsa-miR-145-5p | ARHGAP11B | lncRNA |
| hsa-miR-145-5p | ATP2B1-AS1 | lncRNA |
| hsa-miR-145-5p | C5orf66 | lncRNA |
| hsa-miR-145-5p | C9orf170 | lncRNA |
| hsa-miR-145-5p | CASC9 | lncRNA |
| hsa-miR-145-5p | CBR3-AS1 | lncRNA |
| hsa-miR-145-5p | CCDC144NL-AS1 | lncRNA |
| hsa-miR-145-5p | DNAAF4-CCPG1 | lncRNA |
| hsa-miR-145-5p | GRM5-AS1 | lncRNA |
| hsa-miR-145-5p | HCG18 | lncRNA |
| hsa-miR-145-5p | HELLPAR | lncRNA |
| hsa-miR-145-5p | IQCH-AS1 | lncRNA |
| hsa-miR-145-5p | JPX | lncRNA |
| hsa-miR-145-5p | KCNQ1OT1 | lncRNA |
| hsa-miR-145-5p | LINC00052 | lncRNA |
| hsa-miR-145-5p | LINC00662 | lncRNA |
| hsa-miR-145-5p | LINC00707 | lncRNA |
| hsa-miR-145-5p | LINC00852 | lncRNA |
| hsa-miR-145-5p | LINC01004 | lncRNA |
| hsa-miR-145-5p | LINC01089 | lncRNA |
| hsa-miR-145-5p | LINC01204 | lncRNA |
| hsa-miR-145-5p | LINC01355 | lncRNA |
| hsa-miR-145-5p | LINC01499 | lncRNA |
| hsa-miR-145-5p | LINC01671 | lncRNA |
| hsa-miR-145-5p | LIPE-AS1 | lncRNA |
| hsa-miR-145-5p | MAGI2-AS3 | lncRNA |
| hsa-miR-145-5p | MALAT1 | lncRNA |
| hsa-miR-145-5p | MAPKAPK5-AS1 | lncRNA |
| hsa-miR-145-5p | MEG3 | lncRNA |
| hsa-miR-145-5p | MUC20-OT1 | lncRNA |
| hsa-miR-145-5p | OIP5-AS1 | lncRNA |
| hsa-miR-145-5p | OTUD6B-AS1 | lncRNA |
| hsa-miR-145-5p | PAX8-AS1 | lncRNA |
| hsa-miR-145-5p | PTOV1-AS2 | lncRNA |
| hsa-miR-145-5p | PVT1 | lncRNA |
| hsa-miR-145-5p | SLFNL1-AS1 | lncRNA |
| hsa-miR-145-5p | SNHG1 | lncRNA |
| hsa-miR-145-5p | ST8SIA6-AS1 | lncRNA |
| hsa-miR-145-5p | TBC1D3P1-DHX40P1 | lncRNA |
| hsa-miR-145-5p | TUG1 | lncRNA |
| hsa-miR-145-5p | XXYLT1-AS2 | lncRNA |
| hsa-miR-145-5p | ZNRF3-IT1 | lncRNA |
| hsa-miR-181a-5p | ADIRF-AS1 | lncRNA |
| hsa-miR-181a-5p | ALMS1-IT1 | lncRNA |
| hsa-miR-181a-5p | CASC19 | lncRNA |
| hsa-miR-181a-5p | CDKN2B-AS1 | lncRNA |
| hsa-miR-181a-5p | CRNDE | lncRNA |
| hsa-miR-181a-5p | DANT2 | lncRNA |
| hsa-miR-181a-5p | DSCAM-AS1 | lncRNA |
| hsa-miR-181a-5p | ERICD | lncRNA |
| hsa-miR-181a-5p | FENDRR | lncRNA |
| hsa-miR-181a-5p | GABPB1-IT1 | lncRNA |
| hsa-miR-181a-5p | GUSBP11 | lncRNA |
| hsa-miR-181a-5p | HCG11 | lncRNA |
| hsa-miR-181a-5p | HELLPAR | lncRNA |
| hsa-miR-181a-5p | IGFL2-AS1 | lncRNA |
| hsa-miR-181a-5p | INTS6-AS1 | lncRNA |
| hsa-miR-181a-5p | KCNQ1OT1 | lncRNA |
| hsa-miR-181a-5p | LIFR-AS1 | lncRNA |
| hsa-miR-181a-5p | LINC00294 | lncRNA |
| hsa-miR-181a-5p | LINC00641 | lncRNA |
| hsa-miR-181a-5p | LINC00665 | lncRNA |
| hsa-miR-181a-5p | LINC00667 | lncRNA |
| hsa-miR-181a-5p | LINC00847 | lncRNA |
| hsa-miR-181a-5p | LINC00910 | lncRNA |
| hsa-miR-181a-5p | LINC01232 | lncRNA |
| hsa-miR-181a-5p | LINC01355 | lncRNA |
| hsa-miR-181a-5p | LINC01514 | lncRNA |
| hsa-miR-181a-5p | LINC01579 | lncRNA |
| hsa-miR-181a-5p | LINC01806 | lncRNA |
| hsa-miR-181a-5p | LUCAT1 | lncRNA |
| hsa-miR-181a-5p | MALAT1 | lncRNA |
| hsa-miR-181a-5p | MBNL1-AS1 | lncRNA |
| hsa-miR-181a-5p | MEG3 | lncRNA |
| hsa-miR-181a-5p | MEG8 | lncRNA |
| hsa-miR-181a-5p | MIAT | lncRNA |
| hsa-miR-181a-5p | MIR4458HG | lncRNA |
| hsa-miR-181a-5p | MIR4697HG | lncRNA |
| hsa-miR-181a-5p | N4BP2L2-IT2 | lncRNA |
| hsa-miR-181a-5p | NEAT1 | lncRNA |
| hsa-miR-181a-5p | NORAD | lncRNA |
| hsa-miR-181a-5p | OIP5-AS1 | lncRNA |
| hsa-miR-181a-5p | PAX8-AS1 | lncRNA |
| hsa-miR-181a-5p | PSMA3-AS1 | lncRNA |
| hsa-miR-181a-5p | PSMD6-AS2 | lncRNA |
| hsa-miR-181a-5p | PSMG3-AS1 | lncRNA |
| hsa-miR-181a-5p | PVT1 | lncRNA |
| hsa-miR-181a-5p | RUNDC3A-AS1 | lncRNA |
| hsa-miR-181a-5p | SGMS1-AS1 | lncRNA |
| hsa-miR-181a-5p | SLFNL1-AS1 | lncRNA |
| hsa-miR-181a-5p | SNHG1 | lncRNA |
| hsa-miR-181a-5p | SNHG12 | lncRNA |
| hsa-miR-181a-5p | SNHG14 | lncRNA |
| hsa-miR-181a-5p | SNHG5 | lncRNA |
| hsa-miR-181a-5p | SNHG6 | lncRNA |
| hsa-miR-181a-5p | SNHG7 | lncRNA |
| hsa-miR-181a-5p | ST7-AS1 | lncRNA |
| hsa-miR-181a-5p | STARD13-IT1 | lncRNA |
| hsa-miR-181a-5p | SYNJ2-IT1 | lncRNA |
| hsa-miR-181a-5p | THUMPD3-AS1 | lncRNA |
| hsa-miR-181a-5p | XIST | lncRNA |
| hsa-miR-181a-5p | ZEB1-AS1 | lncRNA |
| hsa-miR-181a-5p | ZNF674-AS1 | lncRNA |
| hsa-miR-181a-5p | ZSCAN16-AS1 | lncRNA |
